# *Raoultella ornithinolytica* as a Potential Candidate for Bioremediation of Heavy Metal from Contaminated Environments

**DOI:** 10.4014/jmb.2212.12045

**Published:** 2023-03-26

**Authors:** Laila Ibrahim Faqe Salih, Rezan Omer Rasheed, Sirwan Muhsin Muhammed

**Affiliations:** 1 Department of Medical Laboratory Sciences, College of Sciences, Charmo University, Sulaimaniya 46001, Iraq; 2 Department of Medical Laboratory Analysis, College of Health Sciences, Cihan University Sulaimaniya, Sulaimaniya 46001, Kurdistan Region, Iraq; 3 Department of Biology, School of Science, University of Sulaimaniya, Sulaimaniya 46001, Iraq

**Keywords:** Bioremediation, heavy metals, metal uptake, *Raoultella ornithinolytica*, tolerance

## Abstract

Disposal of waste containing heavy metals into the environment is a major threat to human health and can result in toxic or chronic poisoning in aquatic life. In the current study, metal-resistant *Raoultella ornithinolytica* was isolated from metal-contaminated samples collected from the Tanjaro River, located southwest of Sulaymaniyah, Iraq. *R. ornithinolytica* was identified by partial amplification of 16S rRNA. The uptake potency of heavy metals was assessed using inductively coupled plasma-optical emission spectroscopy (ICP-OES) and indicated that *R. ornithinolytica* removed 67, 89, 63.4, 55.6, 56.5, 65, and 61.9% of Cd, Pb, Cr, Ni, Zn, Co, and Fe, respectively. These removal rates were influenced by temperature, pH, and contact time; at 35°C and pH 5 with a change in the incubation time, the reduction rate improved from 89 to 95% for Pb, from 36.4 to 45% for Cu, and from 55.6 to 64% for Ni. Gene analysis indicated that *R. ornithinolytica* contained *pbrT*, *chrB*, *nccA*, *iroN*, and *czcA* genes, but the *pcoD* gene was absent. Energy-dispersive X-ray spectroscopy (EDS) images showed evidence of metal ion binding on the cell wall surface with different rates of binding. Transmission electron microscopy (TEM) detected different mechanisms for metal particle localization; cell surface adsorption was the main mechanism for Pb, Zn, and Co uptake, while Cd, Ni, and Fe were accumulated inside the cell. The current study describes, for the first time, the isolation of *R. ornithinolytica* from metal-contaminated water, which can be used as an eco-friendly biological expedient for the remediation and detoxification of metals from contaminated environments.

## Introduction

Contamination of the aquatic environment is one of today’s most pressing environmental challenges, since hazardous levels of pollutants cause ecological and human health problems [1]. Heavy metals have contaminated around 40% of the world’s lakes and rivers [2]. One of the most serious environmental concerns is the presence of hazardous heavy metals in aqueous systems as a result of the discharge of untreated metal-containing effluents into water bodies [3]. Many studies have reported that several human health conditions are directly related to metal intoxication that enters the food chain through the water plant ecosystem [4]. Toxicity, in the form of conformational alteration, denaturation and inactivation of enzymes, or destruction of the integrity of cells and organelles, can occur as a result of metal ion competition with, or replacement of, a functional metal [5]. The treatment of heavy metal-contaminated water is a challenging process; the removal of metal ions from aqueous solutions has been intensively studied using approaches that have mainly focused on physical, chemical, and biological technologies which have been developed and optimized to remove and utilize heavy metals from contaminated environments [6]. The common physicochemical treatment processes for metal remediation in water include precipitation, ion exchange, and reverse osmosis [7]. These methods are costly, inefficient when removing heavy metals from large amounts of water, and have limitations such as high energy consumption, non-selectivity, and the use of chemical products [8]. Bioremediation has become an alternative option to conventional remediation technologies; the use of microorganisms such as bacteria and fungi to remove heavy metals has gained a lot of interest in recent years [9]. These organisms can be used for metal remediation by removing, concentrating, and recovering metals from contaminated sites. Therefore, microbe-related technologies can serve as an alternative or complement to conventional methods for metal removal or metal recovery [10]. Indigenous microorganisms have evolved a variety of mechanisms that enable their survival in the presence of toxic metals; these mechanisms include efflux of toxic metals that enter cells via essential metal transporters, enzymatic transformations that decrease metal toxicity [13], biosorption to the cell walls and entrapment in extracellular capsules, and precipitation or oxidation-reduction reactions [14]. The capacity of microorganisms to detoxify metal pollution can be employed for bioremediation. *Raoultella* spp. are indigenous bacteria that are usually found in aquatic environments and soil [15]; *Raoultella* species are aerobic, gram-negative, nonencapsulated bacteria that were previously members of *Klebsiella*, but are now classed as a separate genus. *Raoultella planticola* and *Raoultella electrica* [16] were recently isolated from heavy metal-contaminated sites and were found to harbor the silver-resistant, cadmiu*m/z*inc/cobalt-resistant, and copper-resistant genes *silA*, *czcA*, and *pcoD*, respectively [17]. *Raoultella* spp. have been applied to the remediation of cadmium from contaminated soil [18]. The roles of *Raoultella* spp. in degrading some pollutants, such as pesticides [19] or uranium [20], have been examined by many researchers. Our goal in this study was to identify local metal-resistant microorganisms and characterize the mechanisms of metal uptake by transmission electron microscope and EDS. Further characterization was performed to determine the location of resistance determinants and explain the role of microorganisms in the removal of heavy metals from industrial wastewater. Although *R. planticola* has been isolated from metal-contaminated water in Turkey [21], to the best of our knowledge, this is the first study on isolating and characterizing metal-resistant *R. ornithinolytica* from metal-contaminated water in Iraq and other neighboring countries.

## Materials and Methods

### Isolation of Metal-Resistant *Raoultella* spp.

**Fig. 1:**
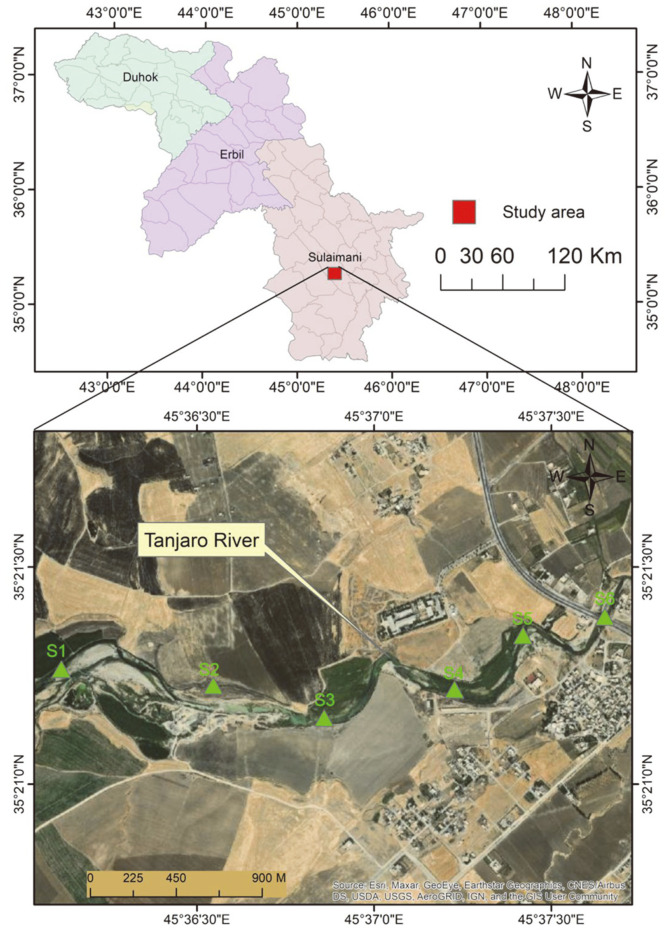
Study area location along the Tanjaro River (Google Maps, 2019).

Samples were collected from the heavy metal-contaminated Tanjaro River located 7 km southwest of Sulaymaniyah, Iraq (35° 16¢35¢¢N 45° 51¢9¢¢E) (Fig. 1). Aseptically collected water samples were serially diluted and inoculated on LB agar medium separately supplemented with 10 ppm of various heavy metal salts (CdSO_4_·4H_2_O, Pb(CH_3_COO)2·3H_2_O, CuSO_4_, K_2_Cr_2_O_7_, Ni(NO_3_)_2_·4H_2_O, Zn(CH_3_CO_2_)_2_, CoCl_3_·6H_2_O, and FeCl_3_). The cultures were incubated at 35 ± 2°C for 24 h. The suspected colonies were carefully chosen and re-cultured on new media for purification to get a single pure strain. Bacterial candidates were initially screened with the VITEK2 system using a gram-negative kit for primary identification of *Raoultella* spp. as described by the manufacturer (BioMerieux, USA).

### Molecular Identification of Isolated *Raoultella* spp.

The genomic DNA was extracted using a Presto Mini gDNA Extraction Kit (Geneaid Biotech Ltd., Taiwan) according to the manufacturer’s instructions. PCR analysis was performed using universal bacterial 16S rDNA primers from [22]: 7F (5'-AGAGTTTGATYMTGGCTCAG-3') and 1015R (5'-ACGGYTACCTTGTTACGACTT- 3'). The PCR was run using the following conditions: initial denaturation temperature of 95°C for 5 min, 35 cycles of 95°C for 30 s, 60°C for 30 s, and 72°C for 3 min with a final extension temperature of 72°C for 5 min. DNA sequencing was performed by the Sanger method using a 3500xL Genetic Analyzer (Applied Biosystems, USA). Multiple sequence alignment was carried out using the Bio-Edit software program, version 7.2.5. The consensus sequences were aligned and compared with other 16S rRNA genes in GenBank using the NCBI basic local alignment search tool (BLAST) program (http://www.ncbi.nlm.nih.gov/Blast.cgi). The phylogenetic tree of *Raoultella* spp. was created based on the sequences of 16S rRNA using the MEGA X software program, version 10.7.1 [23]. The tree was constructed based on the maximum likelihood method [24].

### Maximum Tolerable Concentrations of Heavy Metals

The MTC was defined as the highest concentration of heavy metals that allowed for development after two days [25, 26]. Using the 96-well microtiter plate technique, the maximum tolerable concentrations (MTCs) of eight different metals were determined. Bacterial isolates were precultured in liquid LB medium for 24 h at 37°C/120 ×*g* until an OD of 0.6 at 600 nm was achieved. Next, 50 μl of the preculture was added to 150 μl of LB broth containing 20 ppm of a separate heavy metal compound as a starter. The mixture was then transferred to a 96-well microplate and incubated for 48 h at 37°C/120 ×*g*; a microplate reader was used to determine the maximum tolerated concentration. For multiple resistance, *Raoultella* spp. were grown in LB medium integrated with a solution of the eight metals collectively in equal concentrations of (1:1:1:1:1:1:1:1 ppm) with the pH adjusted to 7.0 and incubated at 35 ± 2°C/120 ×*g* for 24 h. After incubation, the resistance potential of multiple heavy metals was assessed [11].

### Determination of Heavy Metal Removal Efficacy

In a batch experiment, *R. ornithinolytica*’s ability to remove heavy metals was evaluated; a 500 ml container containing 200 ml of LB broth was prepared individually for each metal and inoculated with 2 ml of an 18 h-old bacterial culture having an OD_600_ of 0.6. The culture was incubated for 24 h at 37°C/120 ×*g*. The culture was then centrifuged at 5,000 ×*g* for 20 min (Sigma S-16P, Germany). At 100°C, the supernatant was digested with 1:1 HNO_3_: HCl, and inductively coupled plasma - optical emission spectroscopy (ICP-OES) (Optima 7300 V, Thermo Fisher, USA) was used to identify heavy metal concentrations in the medium before bacterium inoculation and following 24 h of incubation .The same treatment without the inoculation of bacterial strains was used as a control for each heavy metal. The results were compared with the control to calculate the heavy metal remediation capacity (%) as follows:

% of heavy metal utilized = heavy metal utilized / heavy metal added to the LB broth ppm ×100

heavy metal utilized = heavy metal added to the LB broth – heavy metal remaining at the end of culture [11, 27].

### Optimization for Heavy Metal Removal by *R. ornithinolytica*

Adsorption and removal of heavy metals by the bacterial cell are influenced by contact time, temperature, and pH. *R. ornithinolytica* was inoculated into LB medium supplemented with the eight metal ions individually according to MTC concentration. Heavy metal removal percentages were calculated for various time intervals (18, 24, 48, and 72 h). Various incubation temperatures (15, 25, 35, and 45°C) were used to determine the optimal temperature for maximal removal of heavy metals. The pH levels used were (5, 7, and 9). These culture conditions were maintained for optimal microbial development [28].

### Effect of Different Heavy Metals on *R. ornithinolytica* Growth

A growth curve experiment was conducted in LB broth. For this purpose, 250 ml flasks containing 100 ml L.B medium were separately supplemented with the heavy metals according to MTC value. Flasks were inoculated with 0.5 ml of overnight culture and incubated in a shaking incubator at 37°C/120 rpm. After 0, 4, 8, 12, 16, 20, 24 and 28 h, growth was monitored by measuring the absorbance at 600 nm using the Spectrum SP-2000UV spectrophotometer, and the growth curves were plotted by the readings obtained from the experiment and compared [11].

### Plasmid Curing

To determine if the resistance genes were encoded on plasmids or chromosome, different concentrations of curing agents, *i.e.*, sodium dodecyl sulfate (SDS) (8, 10, 12% w/v) and ethidium bromide (1.0 to 10 μg/ml) as described by [29] were used. The bacteria cells were incubated in the presence of the curing agents. After incubation, 0.5 ml of the culture was spread on LB agar without heavy metals and with 10 ppm of different heavy metals. After a 24 h incubation at 37°C, the cured cells were detected by comparing the development of bacterial colonies on heavy metal-containing plates with those without heavy metals. The samples that showed colonies on normal LB agar but failed to grow on LB agar supplemented with different heavy metals were the possible cured cells [30].

### PCR Analysis Targeting Genes Encoding Heavy Metal Tolerance

Primers targeting (cadmium, zinc, and cobalt efflux pump) genes *czcA*; copper resistance genes pcoA (copper efflux pump); chromate resistance genes *chrB*, lead resistance gene *pbrT*, nickel resistance gene *nccA*; and iron resistance gene *iroN* were used to amplify metal resistance encoding genes as described in Table 1. The primers amplified 320, 500, 450, 448, 1,141 and 667 base pairs respectively [31]. PCR programs were run under an optimized condition of amplification as summarized in Table 2.

### Field Emission Scanning Electron Microscopy (FE-SEM) and Energy Dispersive X-Ray Spectroscopy (EDS) Analysis

FE-SEM and dispersive X-ray spectroscopy were conducted to characterize *R. ornithinolytica* before and after treatment with heavy metals to detect any changes in cell morphology occurring as a result of metal exposure. The bacterial cultures with and without heavy metals were pre-fixed on a grid with aldehyde (2.5% (v/v) glutaraldehyde) in PBS for 3 h at 4°C, before being post-fixed with 0.5% (v/v) osmium. Samples were then dehydrated in a series of ethanol and hexamethyldisilazane (HMDS) solutions (Sigma, Australia). Following that, the samples were dried in 1:1 ethanol/HMDS, followed by drying twice in 100% HMDS. Subsequently, the dried samples were sputter-coated with gold for 120 s at 22 kV [35]. Samples were then scanned by FE-SEM and EDS, with the accelerating voltage applied at 15 kV for FE-SEM and 20 kV for EDS images [36].

### Determining the Localization and Distribution of Heavy Metals in *R. ornithinolytica*

TEM was used to identify the location of heavy metal particles within the cells. *R. ornithinolytica* was inoculated into LB broth and grown at 37°C with shaking at 120 rpm. Heavy metals were subsequently added to the growth medium according to their minimum inhibitory concentration (MIC) values and cultured for an additional 24 h at 37°C. Cells without any treatment served as the control. The cells were fixed with 3% (v/v) glutaraldehyde, followed by post-fixation with 1% (v/v) osmium tetroxide (OsO_4_) for 2 h and rinsed with PBS. After washing, specimens were dehydrated using a series of ethanol treatments (30, 50, 70, 80, 90, 95, and 100%). The dehydrated specimens were embedded in Spurr resin and incubated for 4 h at 25°C. Polymerization was achieved by incubating the specimens at 65°C for 24 h. The solidified specimens were sectioned and stained with uranyl acetate and alkaline lead, respectively, and examined by TEM (Carl Zeiss, EM10C-100Kv, Germany) using a modified procedure of [37].

## Results and Discussions

### Isolation and Identification of Heavy Metal-Tolerant *R. ornithinolytica*

The toxic effect of metal ions exerts selective pressure on microorganisms whereby bacteria that are resistant to these metals survive [38]. *Raoultella* sp. was isolated from the metal-contaminated Tanjaro River, where the industrial effluents and sewage from the city of Sulaymaniyah, including agricultural and hospital discharges, flow into the river. The overall mean concentrations of the heavy metals in the Tanjaro River were in the following order: Zn > Cu > Pb > Ni > Co > Fe > Cr > Cd, with values of 0.086, 0.073, 0.071, 0.068, 0.051, 0.056, 0.031, and 0.024 ppm, respectively. Some of these concentrations exceeded the WHO water quality criteria [39].

**Fig. 2:**
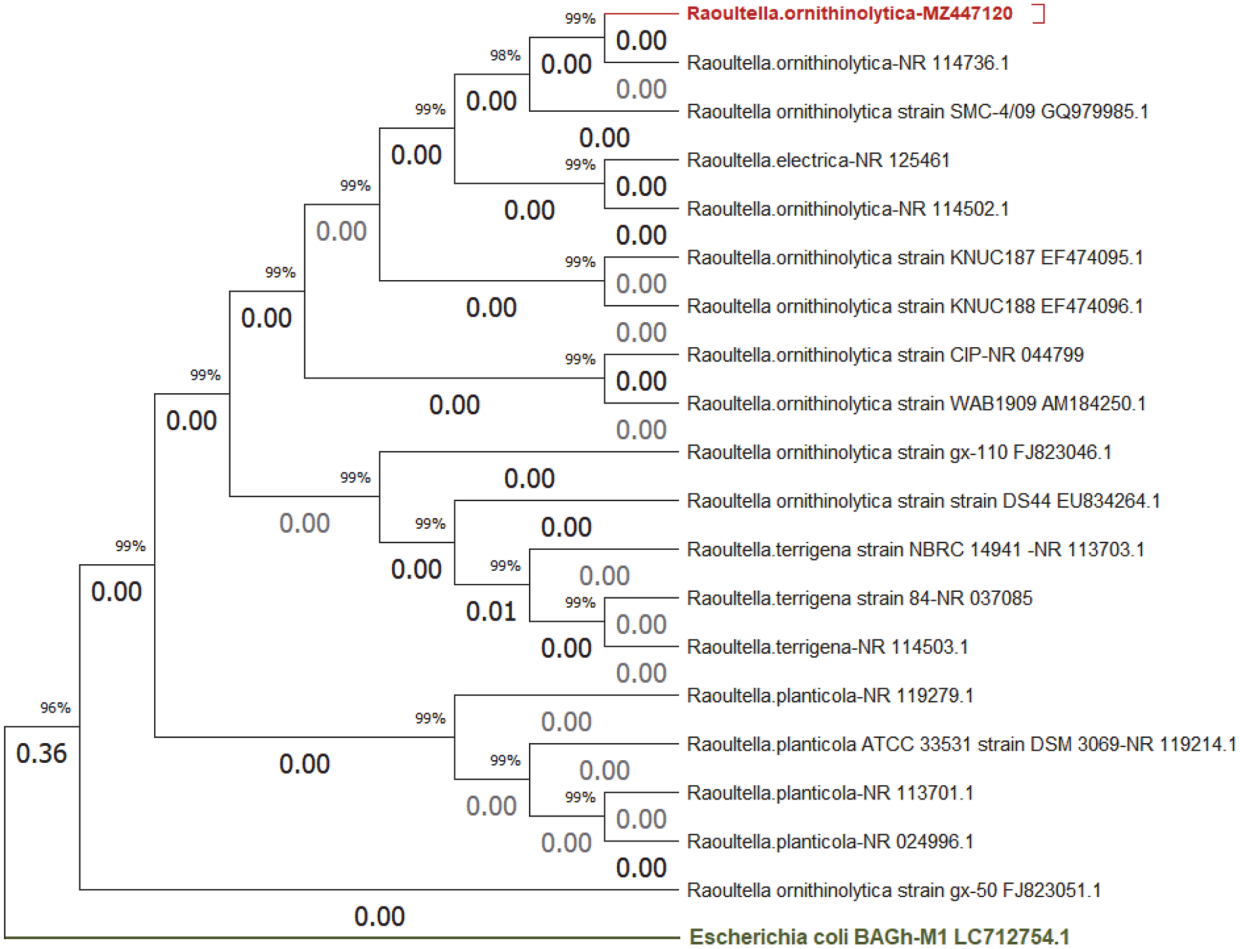
Phylogenetic tree of *R. ornithinolytica*-RO40LCH based on partial 16S rRNA gene sequencing.

The isolated bacteria were identified biochemically and confirmed by molecular tools as *R. ornithinolytica*-RO40LCH with the accession number MZ447120. Phylogenetic analysis based on the 16S rRNA gene sequence was performed. A partial sequence of 16S rRNA (Fig. S1) of isolate RO40LCH was assigned to the species *R. ornithinolytica* (Fig. 2). According to the phylogenetic results, our isolate *R. ornithinolytica*-RO40LCH is most closely related to *R. ornithinolytica* NR_114736.1. *Raoultella* sp. is a gram-negative, rod-shaped, oxidase-negative and catalase-positive bacterium. Many *Raoultella* spp. have been isolated as environmental strains and some have the ability to degrade different organic compounds. A previous study, for example, indicated that *R. planticola* is a promising, polycyclic aromatic hydrocarbon (PAH)-degrading strain with high potential for the remediation of mixed PAH contamination [40]. The current study, however, provides the first description of the isolation of *R. ornithinolytica* from metal-contaminated water in Iraq.

### Evaluation of Heavy Metal Tolerance

The ability of the bacterial isolates to resist different concentrations of heavy metals was evaluated by determining maximum tolerable concentrations (MTCs), which were evaluated as both single and multi-metal tolerances. In the presence of individual metals, *R. ornithinolytica* tolerated Cd, Pb, Cr, Co, and Fe at concentrations of 120, 430, 230, 210, and 340 ppm, respectively.

**Fig. 3:**
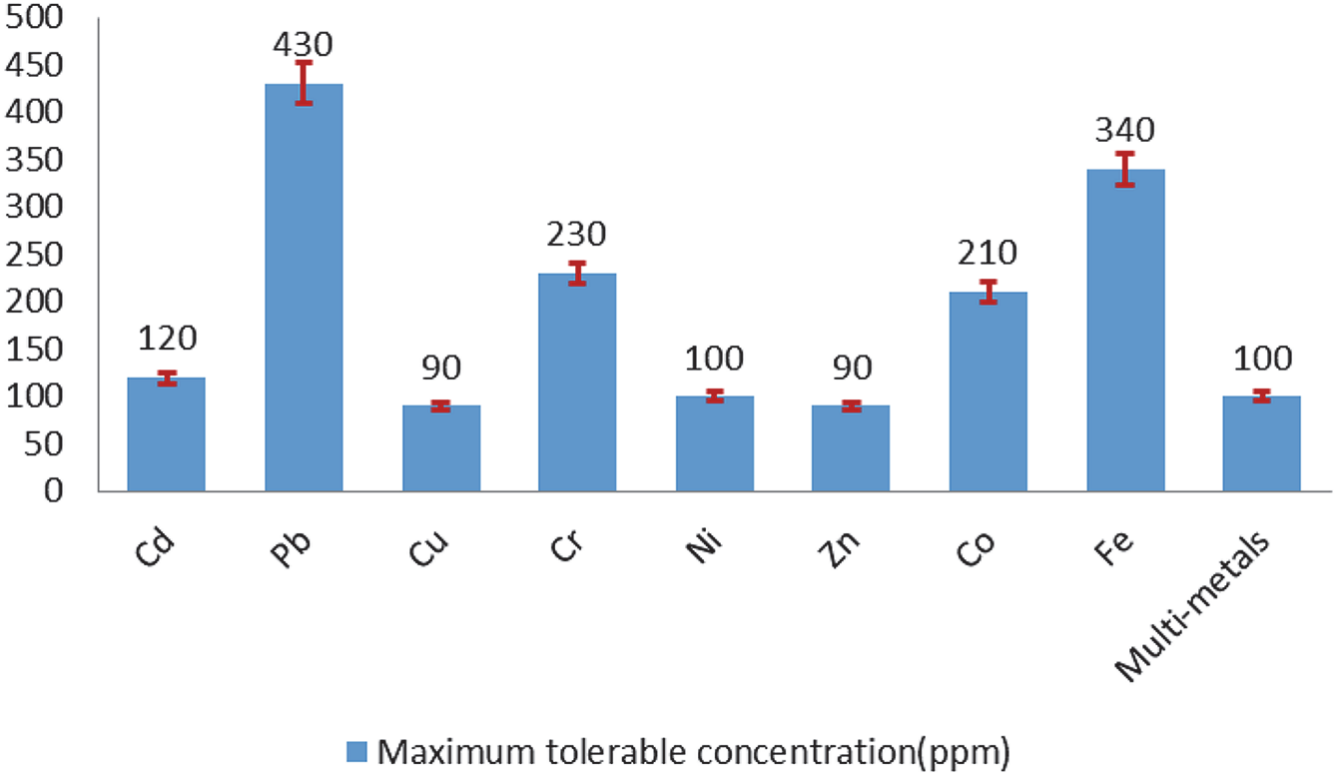
Heavy metals maximum tolerable concentration (MTCs) of *R. ornithinolytica*.

When the metals were combined in the culture, the bacteria tolerated 100 ppm of each of the eight metals collectively (Fig. 3), which is high compared to bacterial metal tolerance reported by others [9, 41]. In our study, Pb was the metal most tolerated by our selected strain, while Cu was the most toxic. Resistance to Fe, Co, Ni, and Cd was intermediate. These results indicate that this strain has a potential application for the bioremediation of heavy metals.

High tolerance variations have been observed for different metals. Exposure to toxic heavy metals gives rise to the development of resistance mechanisms in the microorganism so that microbial populations in metal-polluted environments adapt to toxic concentrations of heavy metals and become metal resistant [13]. In vitro, factors such as the culture media, pH, temperature, and incubation time, as well as the diverse forms and concentrations of metals, may influence the metals' toxicity. Due to these factors, there are no universally accepted metal concentrations to define bacterial tolerance or resistance [42]. A further complication is that variation in metal tolerance might be due to the presence of different tolerance mechanisms [43]. Among the investigated heavy metals in our study, Pb and Fe were the most tolerated, followed by Cr, Co, and Cd, while Cu was the least tolerated. This may be attributable to the high level of heavy metal pollution at the site where the water samples were taken. The study of [44] stated that heavy metal pollution is of particular concern in Iraqi Kurdistan due to the many emission sources, including low-quality petrol, a widespread paint industry, and unsafe disposal into water sources of car batteries and other batteries containing heavy metal products. These results were similar to those reported by [11].

### Heavy Metal Removal Efficacy

**Fig. 4:**
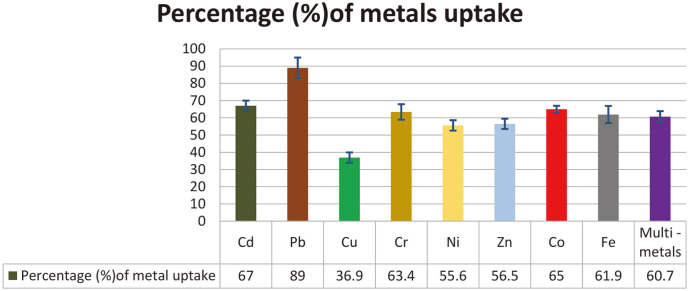
Percentage of heavy metals uptake by *R. ornithinolytica*.

The ability of *R. ornithinolytica* to remove heavy metals from the medium was measured by ICP-OES. The results showed that *R. ornithinolytica* can achieve high-level removal of the metals tested in this study (with the exception of Cu), with reductions of 67, 89, 63.4, 55.6, 56.5, 65, and 61.9% for Cd, Pb, Cr, Ni, Zn, Co, and Fe respectively. Furthermore, 60.7% of the heavy metals were removed when present in combination (Fig. 4), implying that this isolate could be a promising candidate for the practical bioremediation of heavy metal-polluted environments. Although the resistance of *R. ornithinolytica* to different metals followed a particular order (Pb> Fe> Cr > Co > Cd > Ni > Zn > Cu), the relative metal removal levels were different: Pb > Cd > Co > Cr > Fe > Zn > Ni > Cu. This is consistent with results obtained by [45], in which Streptomyces spp. showed high resistance to some metals but a low capacity to remove these metals; indeed, the study stated that there was no correlation between resistance level and accumulation capacity for the tested strains.

### Optimum Condition for Heavy Metal Removal

**Fig. 5:**
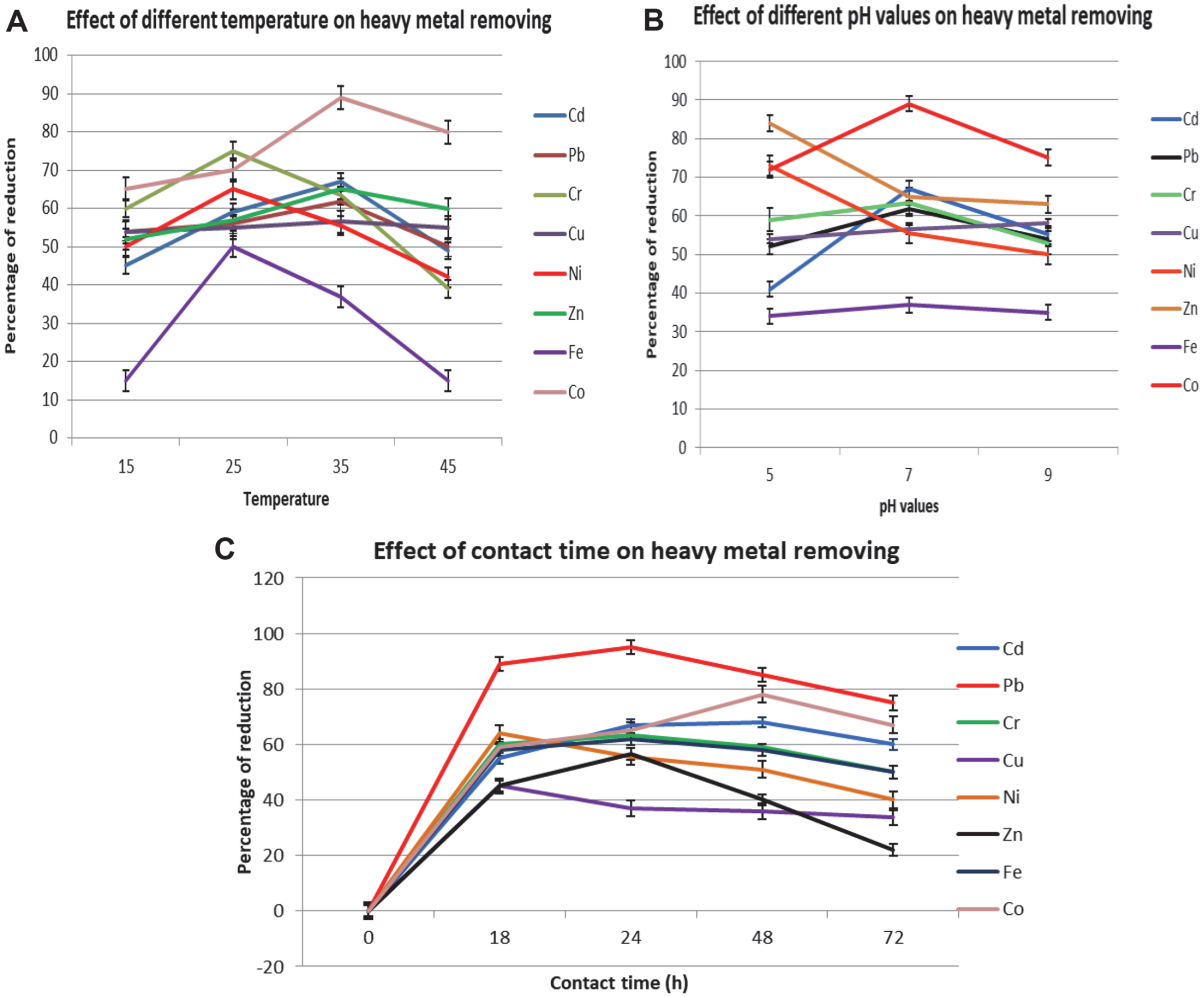
Effect of different environmental condition on heavy metal removing by *R. ornithinolytica*. (**A**) Effect of different temperatures (**B**) Effect of different pH value (**C**) Effect of contact time.

The heavy metal type and concentration, as well as the environmental growth conditions, such as temperature, pH, and the duration of the microorganisms' interaction with toxic metals, all affect the ability of live cells to remove metal ions from aqueous solutions [44]. In this study, we analyzed the effects of temperature, pH, and incubation time on metal uptake by *R. ornithinolytica*. The effect of different incubation temperature on the uptake of the eight selected metals (Fig. 5A) was examined, revealing that 35°C was the optimum temperature for Cd, Pb, Zn, F, and Co uptake. At 35°C, the removal rates were increased compared to 37°C, changing from 45 to 67, 65 to 89, 55 to 56.5, and 50 to 65% for Cd, Pb, Zn, F, and Co respectively. In addition, 25°C was optimum for Cr, Cu and Ni uptake, with maximum rates of metal reduction of 75, 50, and 65% for Cr, Cu, and Ni respectively. These findings are in agreement with the study of [5] who reported that maximum biosorption rates for Cd, Co, and Pb by *Enterobacter* sp. were obtained at 35°C, while the best temperature for Zn and Cu uptake was 25°C. Furthermore, *Arcanobacterium bernardiae* and *B. amylolikuefaciens* have been shown to achieve their maximum capacity for Pb uptake at 35°C [46].

Metal solutions at high temperatures can inhibit or denature enzymes, as well as harm structural components of the plasma membrane, limiting the growth of bacteria and their ability to take up metals from the medium [44]. This can be attributed to a decrease in metabolic activity caused by the increase in temperature above optimum. Conversely, when the temperature falls below the optimum level, bacterial activity is also reduced because most enzymes are less active at low temperatures [42].

The bacterial growth, activity, metal bioaccumulation, and biosorption capabilities are influenced by pH, an important environmental factor that not only affects bacterial activity but also the chemical behavior of metal ions in solution [47]. The results of pH variation indicated that pH values in the range of 7-8 are optimum for the uptake of most of the selected metals (Cd, Pb, Cr, and Fe) (Fig. 5B), which agrees with studies by [5, 48], in which the optimum adsorption of Cd and Cu by *Enterobacter* sp. *Ochrobactrum anthropi* occurred at pH 7–8. The same result was obtained by [49], who suggested that the adsorption of Cd increased with increasing pH due to increased negative surface charges. The adsorption of Cd was influenced by the pH of the aqueous medium, and the adsorbed amount gradually increased with decreasing acidity. The highest removal of Co and Ni was obtained at pH 5 in our study, in agreement with the results obtained by [50], who revealed that maximum metal removal was obtained at pH 6.25.

The contact time between the bacterial cells and the metal solutions is an important factor affecting metal uptake. Fig. 5C shows the uptake of heavy metals by *R. ornithinolytica* over time, from 0-72 h. The maximum removal of Pb, Cu, and Ni was reached after 18 h incubation, at which point their uptake reached 95, 45 and 64%respectively, consistent with results obtained by [51], who reported that the biosorption of Pb by *Phanerochaete chrysporium* was rapid during the initial stages of incubation until equilibrium was reached.

Conversely, the highest uptake for Cr, Zn, and Fe by *R. ornithinolytica* was reached by 24 h. Cu showed only 46%uptake after 72 h incubation. In a study done by [52], it was concluded that the removal capacity of Ni and Cd reached the maximum at 24 h and 48 h, respectively. Similar findings with respect to Cd biosorption were reported by [53].

The analysis of the effect of contact time on metal uptake revealed that each heavy metal had an optimum period, and once this time had passed, uptake remained steady or slightly decreased. This agrees with metal uptake models, where the process can be considered as an equilibrium that involves adsorption and desorption due to saturation; as a result, exposing tested organisms to metal ions for longer than the optimum time may not improve metal uptake [54]. The optimal conditions for improving the remediation efficiency of each metal are listed in Table 3.

### Effect of Heavy Metals on Growth of *R. ornithinolytica*

**Fig. 6:**
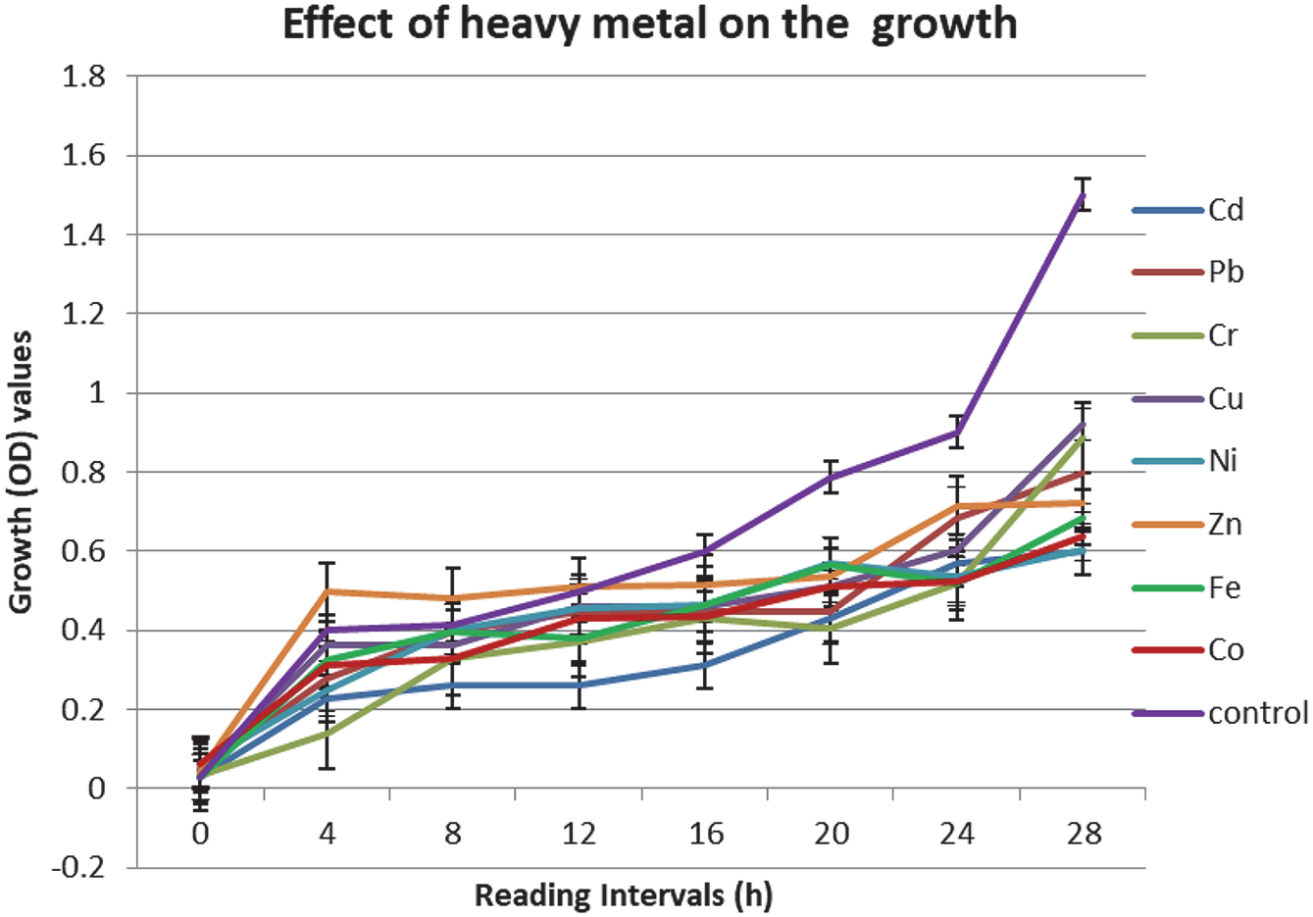
Effect of heavy metal on the *R. ornithinolytica* growth.

The presence of heavy metals acts as a stress on the bacteria, causing an overall reduction in bacterial growth. Fig. 6 shows the effect of heavy metals on the growth of *R. ornithinolytica* compared to its respective growth patterns in the absence of heavy metal addition. The growth in medium containing heavy metals was slower than that in medium without metal addition, which may attributable to the toxic effects of heavy metals that inhibit the growth and reproduction of some bacteria and reduced their biomass if they reached concentrations above the tolerance level of the bacteria [55]. The presence of lead had little impact on the growth rate of *R. ornithinolytica*, which might be due to a well-developed lead tolerance mechanism occurring as a result of high Pb concentrations in the Tanjaro River. The lower concentration of Cd in the water samples, meanwhile, resulted in a lower tolerance rate for this metal ion and a slower growth rate than in the absence of heavy metal, similar to findings observed by [56].

### Plasmid Curing

**Fig. 7:**
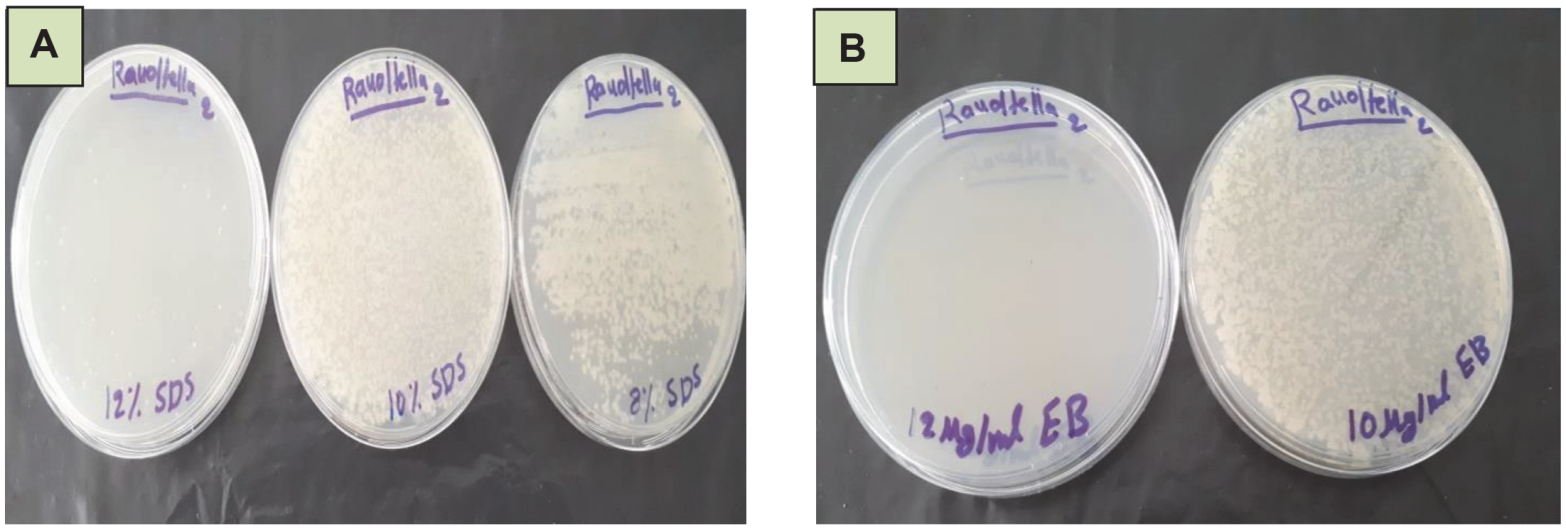
Plasmid curing of *Raoultella ornithinolytica* in medium supplemented with different concentrations of A- SDS, B- E.B.

Heavy metal resistance could be mediated by genes on chromosomes, plasmids, or transposons; the plasmids carried genes responsible for resistance to high levels of toxic heavy metals [57]. In the current study, SDS and ethidium bromide (EB) were separately used as curing agents, and after a 24 h incubation at 37°C, the capacity of living cells to remove metal ions from aqueous solutions was detected comparing the development of bacterial colonies on heavy metal containing plate with that of the normal (without heavy metals) plate as shown in (Fig. 7). The ability of *R. ornithinolytica* to grow in the presence of different heavy metals was plasmid encoded and this ability is lost after treating the bacteria with 12% SDS or 10 μg/ml E.B. The study by [58] reported that the frequency of the occurrence of plasmids in heavy metal resistant bacteria was more than that in common bacteria. Similar results were concluded by [5], who worked on the ability of *Enterobacter* sp. for metal uptake from polluted industrial waste water in Egypt.

### Metal Resistance Genes

**Fig. 8:**
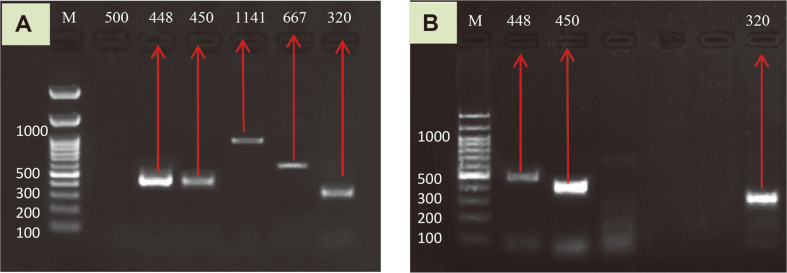
Agarose gel electrophoresis of metal resistant genes in *Raoultella* sp. (**A**) R. ornitholytica; (**B**) *R. planticola*. M= DNA ladder (100bp); lanes **1**- *pcoD* gene 500bp, **2**- *pbrT* gene 448pb, **3**- *chrB* gene450pb, **4**- *nccA* gene 1141 pb, **5**- *iroN* gene 667 pb, **6**- *czcA* gene 320 bp.

*Raoultella ornithinolytica* isolated from Tanjaro River water had high level of resistance to the selected eight heavy metals, and it is clear from the results that the bacterium showed good absorption/ adsorption potential. In the bioremediation processes, heavy metal resistance genes are of great importance. Heavy metal resistance bacteria (HMRB) bearing multiple heavy metal-resistant genotypes and phenotypes could be more promising in bioremediation applications in complex environments [59]. Bacterial resistance to heavy metals is a complex process and the mechanisms involved are mainly transportation, biosorption, and co-metabolism/ redox, which are determined by many genes at the genetic level. For instance, *czcA* (cadmium, zinc, and cobalt efflux pump) and *chrB* efflux protein have been found for the transportation of chromium, while *pbrT* is responsible for the biosorption of Pb, and *pcoD* is related to the copper efflux pump [60, 61]. The occurrence of heavy metal tolerance genes in *Raoultella* sp. isolated from wastewater samples is depicted in Figs. 8A and 8B. *R. ornithinolytica* contains five genes out of the six selected metal resistant genes (*pbrT*, *chrB*, *nccA*, *iroN*, and *czcA*) which are responsible for (Pb biosorption, Cr efflux, Ni/Co efflux protein, iron uptake and Co/Zn/Cd efflux) and consist of 448, 450, 1141,667 and 320 bp, respectively; *pcoD* gene, which is responsible for copper efflux, was absent, which may be the reason for *R. ornithinolytica*’s lower resistance for copper in comparison to the other metals [17]. This could be because copper is widely used in hospitals, especially in surfaces for preventing biofilm formation and healthcare-associated infections. The Tanjaro River is located far away from any hospital, which may be the reason for the low copper concentration in the water and low resistance. Meanwhile, *R. planticola* contains three genes out of six (*pcoD*, *pbrT*, *czcA*), and a study done by [22] found that *R. planticola* was resistant to each of copper, iron, lead, manganese, and nickel. Multiple heavy metal-resistant phenotypes were identified with a higher rate of resistance and bioremediation potential in this study, and this was not reported in previous studies. Although there were inconsistencies between heavy metal-resistant phenotypes and genotypes, as only 5 metal resistance genes were detected in *R. ornithinolytica*, it phenotypically showed resistance to the eight selected metals, meaning that this HMRB strain could potentially provide a gene pool for future genetic methods for metal bioremediation.

### Field Emission Scanning Electron Microscopy (FESEM) and Energy Dispersive X-Ray Spectroscopy (EDS) Analysis

Field emission scanning electron microscope (FE-SEM) was used to detect any change in the morphology of the cells as a result of metal exposure and normal *R. ornithinolytica* without metal stress (control) was compared with metal stress to see the surface changes in bacteria due to metal stress.

**Fig. 9:**
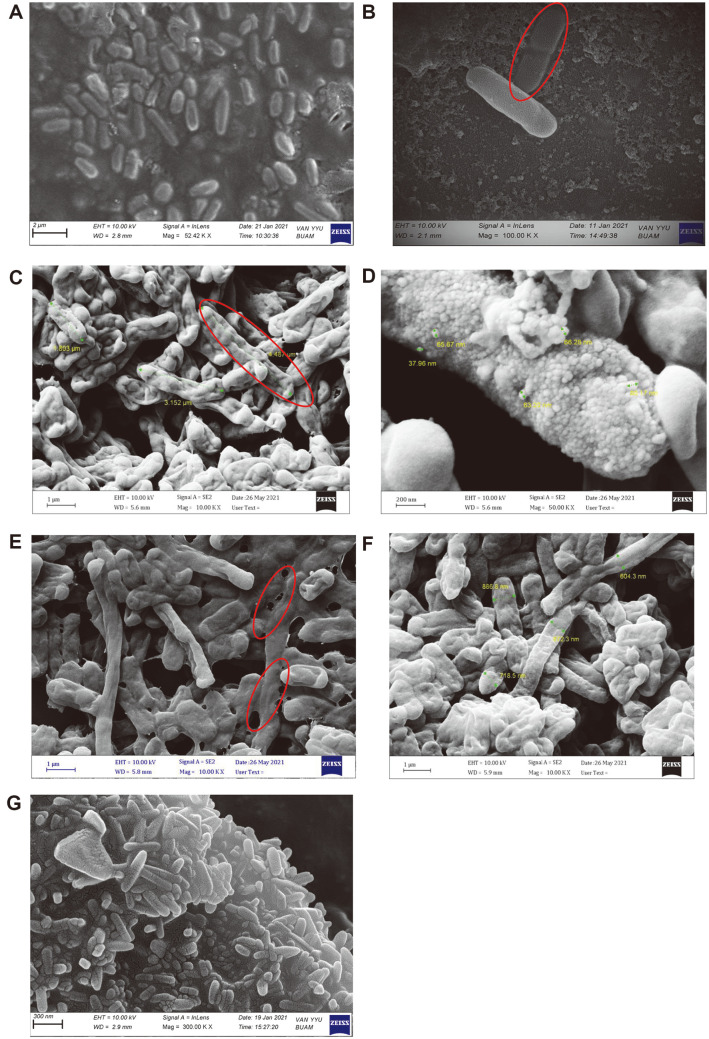
Field emission scaning electron microphage of *R. ornithinolytica* showing the effect of metal stress on the cell morphology and dimension in the (A, B) absence of metal (control); and the presence of (C) Cd; (D) Pb; (E) Cu; (F) Cr; (G) the presence of multi-metals.

The results of *R. ornithinolytica* FE-SEM images (Figs. 9A and 9B) showed that they exist as aggregate short rods or as single cells in untreated culture (control) with some dividing cells being found in the fields under microscope, while the SEM results of the cells cultured with different heavy metal separately revealed changes in the bacterial cell size and the morphology in comparison to the control cells.

Generally, cells when grow under metal stress aggregate and stack on the top of each other and show curvature or a dented appearance. This agreed with [62], which revealed three different types of changes in the cell size and surface morphology in comparison with the control cells. Cells growing in the presence of Cd ion show a decrease in the area/volume ratio, making the cells more elongated and causing them to take on a filamentous appearance, reaching a length of 4.487 μm (Fig. 9C). The same finding was documented by [63], who observed cell surface modifications from smooth to rough and membrane indentations in the presence of metal ions.

Cells in Pb-rich medium clearly show the adsorption and the accumulation of Pb particles on the cell surface (Fig. 9D) with the decrease in the cell size to the nano scale. The same results were obtained by [16], who observed significant accumulation on the surface of LAB treated with lead ions. Fig. 9E represents bacterial cells grown in medium supplemented with copper (Cu), where the morphology of the cell changes to resemble a fuzzy coat around the outer surface, which could be due to additional polysaccharide secretion by the cell, which can reduce the surface area of contact between the cell and metal, thereby preventing further uptake. These same changes were observed by [64], which may explain the reason behind the low resistance and uptake ratio of Cu by *R. ornithinolytica*. In the case of bacterial growth in the presence of eight metals collectively (multi-metal growth), the cells produce a high rate of aggregation that makes the cells difficult to distinguish, with the appearance of cracks on the cell wall, as shown in Fig. 9G.

**Fig. 10:**
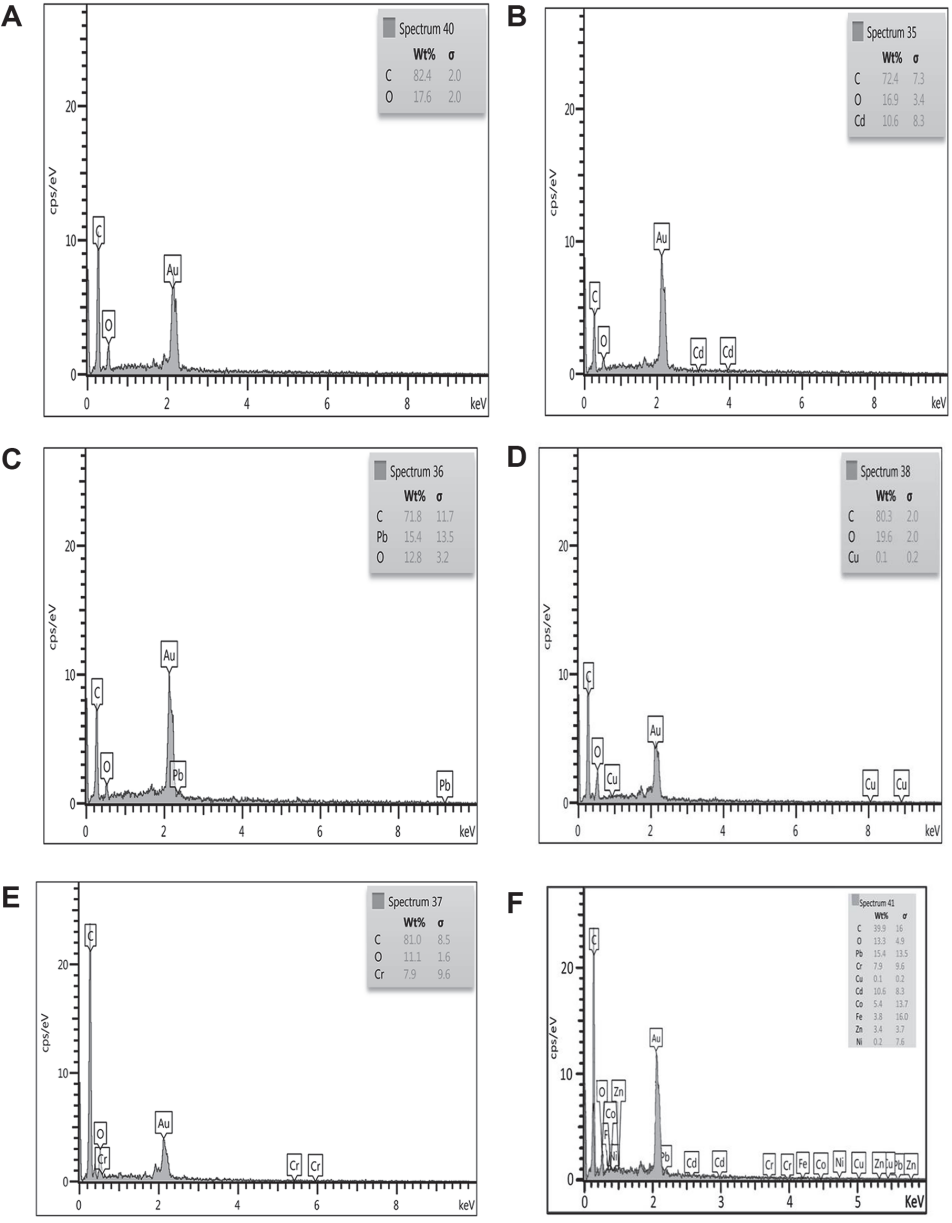
Energy dispersive X-ray spectroscopic (EDS) analysis for elemental composition on the cell surface of *R. ornithinolytica* (A) without metal loading (control) (B) Cd; (C) Pb; (D) Cu; (E) Cr; (F) presence of multi-metals.

On the other hand, energy dispersive X-ray spectroscopy (EDS) analysis was carried out to confirm the presence of different metals besides the other constituent groups of the bacterial cell wall (Fig. 10). EDS spectral images revealed visible evidence of binding metal ions on the cell wall of bacterial cells, which clearly showed that Cd, Pb, and Cr ions were adsorbed on the surface with different rates of binding for different metals. Among the metals, lead was found in major proportion in the cell wall with a weight percentage of 15.4wt% (Fig. 10C). In comparison to the other metals, this confirmed the higher rate of Pb reducing from the medium by bacteria containing *pbrT* genes, which are responsible for the lead adsorption to the cell wall, and Cu showed the minimum amount at 0.1wt%.

### Localization and Distribution of Heavy Metals in *R. ornithinolytica*

**Fig. 11:**
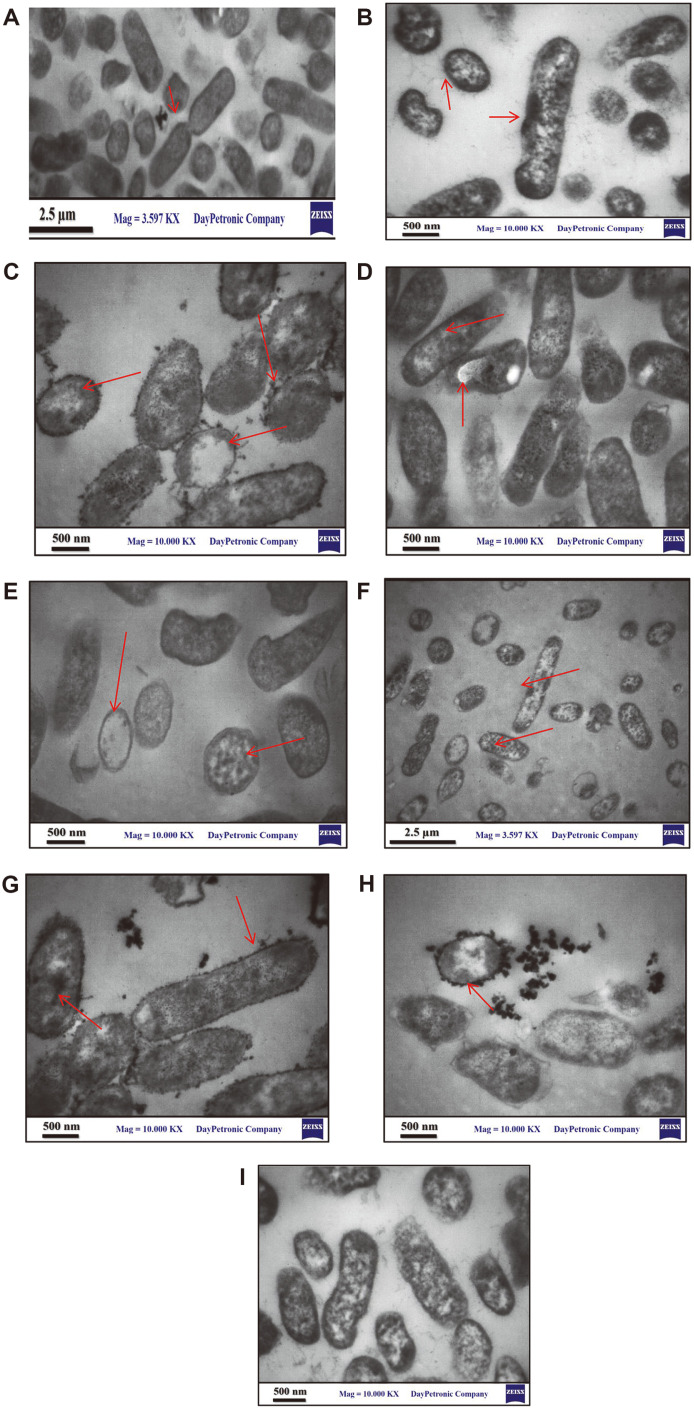
Transmission electron micrograph of *R. ornithinolytica* cultured with different heavy metals; (A) control without any metals; (B) Cd; (C) pb; (D) Cu; (E) Cr; (F) Ni; (G) Zn; (H)Co; (I) Fe.

Transmission electron microscope (TEM) provided an insight into the intra-cellular accumulation of heavy metals, as each of the control and treated cultures were examined. The TEM images (Fig. 11) showed that many electron-dense granules were found, mostly on cell walls and cytoplasmic membrane, [65] suggesting that those electron-dense granules were heavy metal complexes with substances binding heavy metals in the bacterial cell. *R. ornithinolytica* utilizes different mechanisms to uptake different types of metals, and these differences may be due to differences in the cell wall structures, as well as the production of metal binding proteins (metalloproteins). The same results were obtained by [66], who demonstrated that cell wall structure is a key factor in heavy metal uptake. In Pb, Zn, and Co uptake, the granules are mainly found on the cell wall and cell membrane that make cell surface adsorption the candidate mechanism (Figs. 11C, 11G, 11H), while Cd, Ni and Fe were accumulated inside the cell (Figs. 10B, 10F, 10I). The same finding was reported by [67], who worked on metal uptake by *Comamonas* from the Tanjaro River, and [64], who reported the accumulation of Cu and Zn within the cells of *Cupriavidus necator* strain. Only a few studies have reported the participation of *Raoultella* sp. in the metal uptake from the environment. In the present study, living cells of *Raoultella* sp. gave satisfactory metal accumulating results, especially for lead. More studies should be designed at the molecular level to evaluate and identify mechanisms involved in the association between metal resistances and accumulation, while also experimenting with different culture conditions and medium types to upgrade the bioremediation potential of these strains.

## Conclusion

The main findings of this work are: (1) For the first time in Iraq and the Kurdistan region, *R. ornithinolytica* was isolated from the metal-polluted Tanjaro River, indicating that the river is contaminated by heavy metals and can provide promising candidates for practical heavy metal bioremediation applications. (2) *R. ornithinolytica* showed considerable tolerance ability against the studied heavy metals with maximum resistance for lead ion. (3) The strain has the ability to remove all the eight metals selected in this study with the exception of Cu. (4) *R. ornithinolytica* also has the ability to remove 89% of lead from the medium and this could be enhanced by changing the environmental condition to reach 95%, making it an effective agent for lead uptake from lead-contaminated sites. (5) Multiple heavy metal resistance genotypes and phenotypes were found in all the sequenced HMRB genomes, indicating that bioremediation using bacteria isolated in situ may be more efficient.

## Supplemental Materials

Supplementary data for this paper are available on-line only at http://jmb.or.kr.

## Figures and Tables

**Table 1:** List of primers, all primers.

Target gene	Primer (Forward and Reverse)	Gene fragment size (bp)	References
16SrRNA	F- AGAGTTTGATYMTGGCTCAG R- ACGGYTACCTTGTTACGACTT	1401	21
czcA	F- GTTCACCTTGCTCTTCGCCATGTT R- ACAGGTTGCGGATGAAGGAGATCA	320	31
pcoD	F- CTGGCCACACTTGCCTGGGG R- CACGCTACGGCGCCCAGAAT	500	32
pbrT	F-AGCGCGCCCAGGAGCGCAGCGTCTT R- GGCTCGAAGCCGTCGAGRTA	448	31
chrB	F- GTCGTTAGCTTGCCAACATCR- CGGAAAGCAAGATGTCGATCG	450	31
nccA	F- ACGCCGGACATCACGAACAAG R- CCAGCGCACCGAGACTCATCA	1141	33
iroN	F- AAGTCAAAGCAGGGGTTGCCG R- GACGCCGACATTAAGACGCAG	667	34

**Table 2:** Thermocycler PCR condition for detecting heavy metal resistance genes.

Reaction	Cycling conditions				
	Initial denaturation	Denaturation	Annealing	Extension	Final extension
Gene (*chrB*)	94°C 5 min	94°C 30 s	58°C 30 s	72°C 30 s	72°C 5 min
Gene (*nccA*, *iroN*)	94°C 5 min	94°C 30 s	60°C 30 s	72°C 30 s	72°C 5 min
Gene (pcoA, *czcA*, *pbrT*)	94°C 5 min	94°C 30 s	62°C 30 s	72°C 30 s	72°C 5 min
Number of cycles (30)					

**Table 3:** Optimal remediation conditions for each heavy metal.

Heavy metals	pH	Temperature	Contact time
Cd	7	35	48
Pb	7	35	18
Cr	7	25	24
Cu	7	25	72
Ni	5	25	18
Zn	9	35	24
Fe	7	35	24
