# Supplementary material for: Raoultella ornithinolytica as a Potential Candidate for Bioremediation of Heavy Metal from Contaminated Environments
Source: J Microbiol Biotechnol. 2023 Mar 26;33(7):895–908. doi: 10.4014/jmb.2212.12045 (PMC10394344; doi:10.4014/jmb.2212.12045)

File: 25_1510R.ab1 Run Ended: 2020/6/11 23:28:13 Signal G:609 A:806 C:1353 T:1296

Sample: 25_1510R Lane: 1 Base spacing: 17.124107 1073 bases in 12887 scans

10 20 30 40 50 60 70 80 90 100 110 120

TT CGGGGTGAGTTCTCTGTGCGTGAGCGCGCTCCCATAAGGTGTAAGCTACCTACTTCTTTTGCGACCCACTCCCATGGGGTGACGGGCGGTGTGTACAAGGCCCGGGAACGTATTCACCGTAGCATT

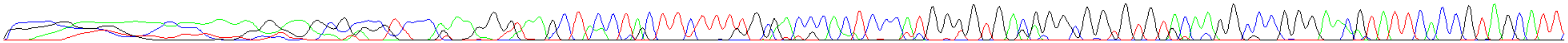

130 140 150 160 170 180 190 200 210 220 230 240 250

CTGATCTACGATTACTAGCGATTCCGACTTCATGGAGTCGAGTTGCAGACTCCAATCCGGACTACGACGCACTTTATGAGGTCCGCTTGCTCTCGCGAGGTCGCTTCTCTTTGTATGCGCCATTGT

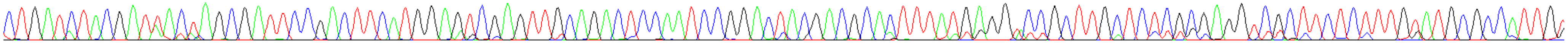

260 270 280 290 300 310 320 330 340 350 360 370 380

AGCACGTGTGTAGCCCTACTCGTAAGGGCCATGATGACTTGACGTCATCCCCACCTTCCTCCAGTTTATCACTGGCAGTCTCCTTTGAGTTCCCGGCCGAACCGCTGGCAACAAAGGATAAGGGTT

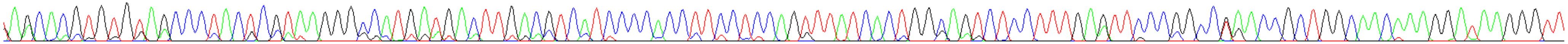

390 400 410 420 430 440 450 460 470 480 490 500

GCGCTCGTTGCGGGACTTAACCCAACATTTCACAACACGAGCTGACGACAGCCATGCAGCACCTGTCTCAGAGTTCCCGAAGGCACCAATCCATCTCTGGAAAGTTCTCTGGATGTCAAGAGTAGG

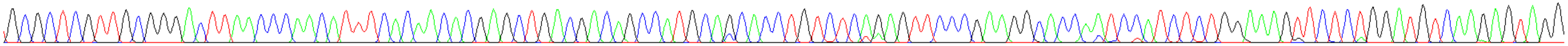

510 520 530 540 550 560 570 580 590 600 610 620 630

TAAGGTTCTTCGCGTTGCATCGAATTAAACCACATGCTCCACCGCTTGTGCGGGCCCCCGTCAATTCATTTGAGTTTTAACCTTGCGGCCGTACTCCCCAGGCGGTCGACTTAACGCGTTAGCTC

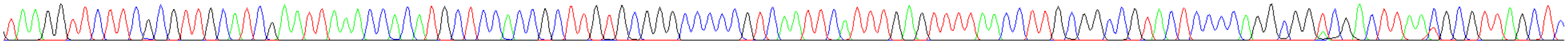

File: 25_1510R.ab1   *Run Ended: 2020/6/11 23:28:13*   *Signal G:609 A:806 C:1353 T:1296*

Sample: 25_1510R   *Lane: 1*   *Base spacing: 17.124107*   *1073 bases in 12887 scans*

640 650 660 670 680 690 700 710 720 730 740 750

CGGAAGCCACGCCTCAAGGGCACAACCTCCAAGTCGACATCGTTTACGGCGTGGACTACCAGGGTATCTAATCCTGTTTGCTCCCCACGCTTTCGCACCTGAGCGTCAGTCTTTGTCCAGGAG

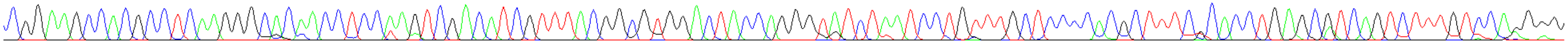

760 770 780 790 800 810 820 830 840 850 860 870

GCCGCCTTCGCCACCGGTATTCCTCCAGATCTCTACGCATTTCACCGCTACACCTGGAATTCTACCCCCCTCTACAAGACTCTAGCCTGCCAGTTTCGAATGCAGTTCCCAGGTTGAGCCCGG

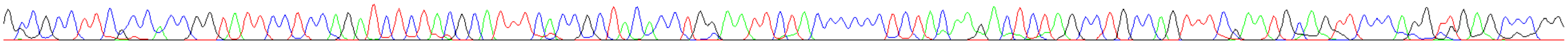

880 890 900 910 920 930 940 950 960 970 980 990 1000

GGATTTCACATCCGACTTGACAGAACCGCCTGCGTGCGCTGTACGCTCAGTAATTCCGATTAACGCTTGCACCCTCCGTATTACCGCGGCTGCTGGCACGGAACTTAGCCGATGCTTCTTCTGC

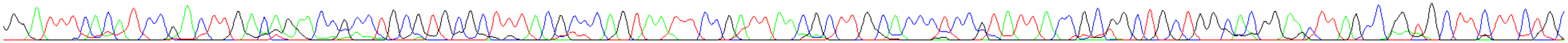

1010 1020 1030 1040 1050 1060 1070

TGGTAACGGCAATCGCCGAGGGGATTAGCCTCAACGCCTTCCTCCCCGCTGAAAGTACTTAAGATCTCCACG

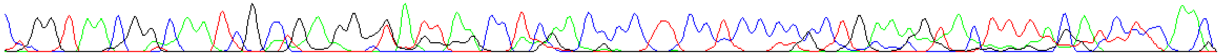

Supplement: Supplementary file 1 [file jmb-33-7-895-supple.pdf]
